# Short exposure to cold atmospheric plasma induces senescence in human skin fibroblasts and adipose mesenchymal stromal cells

**DOI:** 10.1038/s41598-019-45191-2

**Published:** 2019-06-17

**Authors:** Marion Bourdens, Yannick Jeanson, Marion Taurand, Noémie Juin, Audrey Carrière, Franck Clément, Louis Casteilla, Anne-Laure Bulteau, Valérie Planat-Bénard

**Affiliations:** 1 STROMALab, Université de Toulouse, CNRS, EFS, ENVT, Inserm U1031, 4bis Av. Hubert Curien, 31100 Toulouse, France; 2 IGFL, UMR5242, ENS Lyon, 46 Allée d’Italie, 69007 Lyon, France; 3 IPREM, UMR 5254, Université de Pau et des Pays de l’Adour, 64000 Pau, France

**Keywords:** Cell biology, Senescence

## Abstract

Cold Atmospheric Plasma (CAP) is a novel promising tool developed in several biomedical applications such as cutaneous wound healing or skin cancer. Nevertheless, *in vitro* studies are lacking regarding to CAP effects on cellular actors involved in healthy skin healing and regarding to the mechanism of action. In this study, we investigated the effect of a 3 minutes exposure to CAP-Helium on human dermal fibroblasts and Adipose-derived Stromal Cells (ASC) obtained from the same tissue sample. We observed that CAP treatment did not induce cell death but lead to proliferation arrest with an increase in p53/p21 and DNA damages. Interestingly we showed that CAP treated dermal fibroblasts and ASC developed a senescence phenotype with p16 expression, characteristic morphological changes, Senescence-Associated β-galactosidase expression and the secretion of pro-inflammatory cytokines defined as the Senescence-Associated Secretory Phenotype (SASP). Moreover this senescence phenotype is associated with a glycolytic switch and an increase in mitochondria content. Despite this senescence phenotype, cells kept *in vitro* functional properties like differentiation potential and immunomodulatory effects. To conclude, we demonstrated that two main skin cellular actors are resistant to cell death but develop a senescence phenotype while maintaining some functional characteristics after 3 minutes of CAP-Helium treatment *in vitro*.

## Introduction

Cutaneous wound healing and the management of complicate wounds resulting in defects or delays in healing represent a major concern for healthcare systems. Thus skin regeneration is one of the current challenges to address for future research and technology^1^. Normal wound healing is commonly described as four overlapping and coordinated phases represented by hemostasis, inflammation, proliferation, and remodeling^2^. This highly dynamic repair program requires the resolution of one phase to properly proceed into the following one. A complex interplay of epithelial, mesenchymal, vascular and stem cells in concert with tissue-resident or recruited immune cells is involved. Even though this process generally leads to a restoration of tissue integrity and barrier function it is also imperfect as it results in scar formation. Inflammatory cells play a pivotal role thus directing the outcome of the healing response^3,4^ and conditioning fibroblast activation into myofibroblast that produces the fibrotic extracellular matrix within the dermis. Overall mesenchymal stromal cells from the deep dermis including fibroblasts and adipose mensenchymal stromal cells (ASC) are largely recruited during wounding and proposed to actively contribute to skin repair and regeneration^5^. ASC act through various mechanisms including secretion, immunomodulation, as well as remarkable adaptive interplay with dermal fibroblasts that significantly improve the healing issue after injury^6,7^. ASC can also differentiate into adipocytes, an abundant cell type in the dermis, known to support skin homeostasis and regeneration. Adipocytes are key regulators of hair follicle cycle by stimulating the skin stem cell compartment^8–10^. ASC can also convert into myofibroblasts^11^ and reciprocally dermal myofibroblasts are reported to reprogram into adipocytes^12^. Taken together dermal adipose tissue components including ASC, mature adipocytes and fibroblasts have emerged as important actors of skin homeostasis and skin regeneration^13^.

Cold atmospheric plasma (CAP) applications recently raised interest for skin wound healing providing promising experimental and clinical results^14,15^. CAP treatment is reported to accelerate wound healing, stimulate skin closure particularly of chronic wounds. Their antibacterial activity in infected wounds is also largely documented as a promising alternative for ulcer treatment^16^. Nevertheless the mechanisms of action of CAP on skin components remain to be investigated to fully understand how the various skin cell subsets are affected and respond to CAP exposure^17,18^. Keratinocyte protein expression, migration, proliferation and apoptosis^19–21^ have been reported as well as promoting angiogenesis^22^ *in vitro* and *in vivo*^23,24^. Effects observed on skin cells are largely associated with modulation of inflammatory pathways as well as redox-sensitive pathways due to reactive oxygen and nitrogen species (RONS) generated^18,25–27^. However CAP-generated free radicals may also trigger cell death and skin damage^28,29^. Thus divergent beneficial to deleterious effects of CAP are described highlighting that optimization of CAP parameters, control in reactive species produced and a better understanding of their mechanisms of action are still required to safely consider developing into clinical applications.

The present study aimed to characterize the effect of a short exposure to helium guided CAP (CAP He) on skin cells representing key components in the wound healing process. The study focused on primary fibroblasts and ASC isolated from the same human skin samples and demonstrated that those CAP-treated cells were resisting to death and apoptosis and were entering into a functional senescent phenotype *in vitro*.

## Results

### Differential sensitivity of various human primary cell types to CAP He treatment

**Figure 1:**
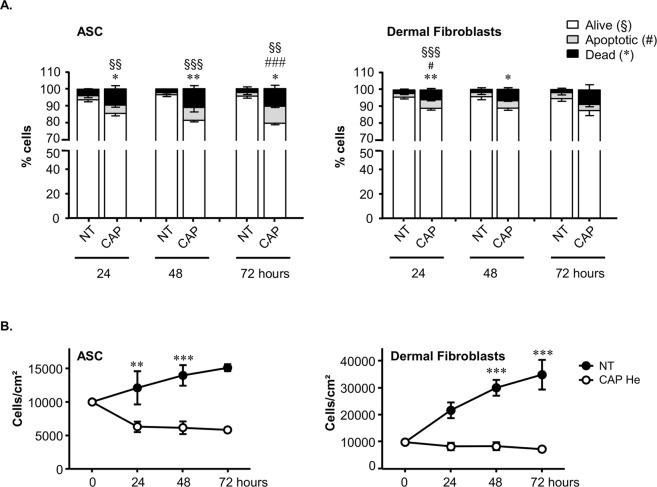
Sensitivity of human ASC and dermal fibroblasts to CAP He treatment. (**A**) ASC and dermal fibroblasts were treated (CAP or NT) and labeled with AnnexinV-APC and Propidium Iodide (PI) at 24, 48 and 72 hours after treatment then analyzed by flow cytometry to determine the percentage of alive, apoptotic and dead cells (n = 3 to 4). (**B**) Cells were treated (CAP or NT) and the number of cell was quantified at indicated times. (ASC, n = 2 to 8; fibroblasts, n = 3 to 7). Data represent mean ± SEM of independent experiments as indicated (n).

Dermal fibroblasts and ASC, relevant cells for skin tissue wound healing, were prepared from the same tissue sample to evaluate their sensitivity to a CAP treatment. Based on a previous comparative study where reactive species produced (nature and quantity) were characterized, a 100% helium gas was chosen to generate CAP^28,30^. For all experiments cells were exposed to CAP for 3 minutes in PBS then maintained in treated PBS for 1 hour at 37 °C before changing for culture medium. Apoptosis and necrosis of both cell types were measured at 24 hours showing that cumulated apoptosis and necrosis slightly increased in CAP-treated dermal fibroblasts and ASC representing 10 to 11% among which 5% corresponded to apoptotic cells (Fig. 1A). After 48 and 72 hours cumulated dead cells slightly increased in CAP treated ASC (18,6% ± 3,9 and 20,8% ± 2,3 respectively) and remained stable in fibroblasts (10,4% ± 1,3 and 12,7% ± 3,9 respectively). To assess if this resistance to death was cell specific, we exposed other freshly prepared human cells, i.e. keratinocytes and immune cells, as control (Supplementary Fig. 1A). Comparatively T lymphocytes turned to be the most sensitive cells to such CAP treatment with more than 90% of cell death after 24 hours, whereas primary keratinocytes were poorly affected by CAP treatment as less than 10% of cell death was induced. Macrophages were also resistant to cell death. We noticed that almost 20% of inflammatory M1 macrophages but not alternatively activated M2 macrophages entered into apoptosis (Supplementary Fig. 1B). Similar results were obtained at 48 and 72 hours (data not shown). Thus this 3 minutes CAP treatment could induce cell death or survival depending on cell type tested.

### CAP He induces a rapid and sustained cell cycle arrest in ASC and dermal fibroblasts

**Figure 2:**
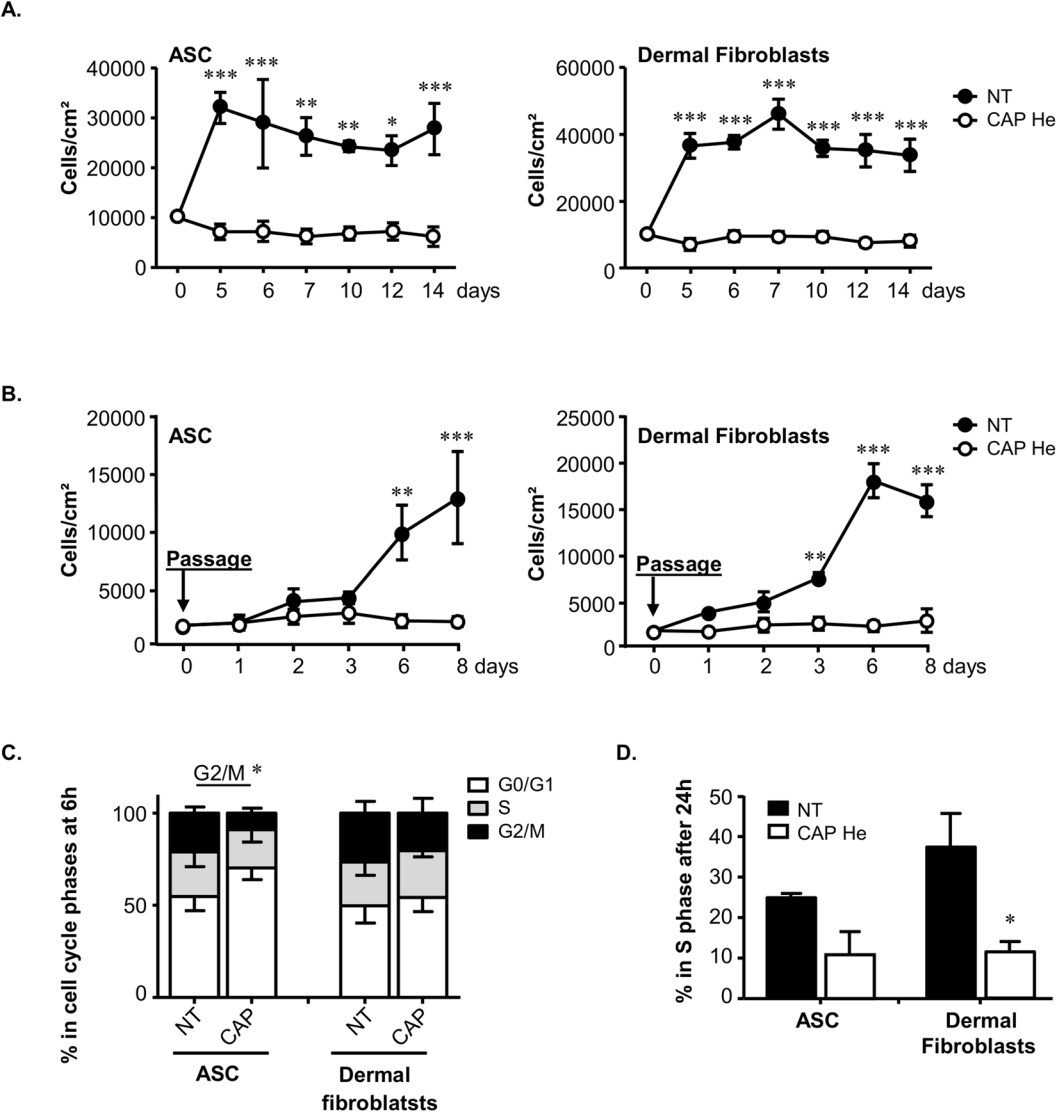
CAP He treatment leads to irreversible proliferation stop and cell cycle arrest. (**A**) ASC and dermal fibroblasts were treated (CAP or NT) and cell number was determined during a longer time-course (n = 3). **(B)** A time-course of cell numeration was performed on treated cells cultured 7 days and passaged once (n = 3). **(C)** Cell cycle analysis was performed by Propidium Iodide staining and flow cytometry to estimate the distribution in G0/G1 and G2/M 6 hours after treatment (ASC, n = 3; fibroblasts, n = 4). **(D)** The percentage of cells in S phase 24 hours after treatment was determined after EdU incorporation by flow cytometry (ASC, n = 3; fibroblasts, n = 4). Data represent mean ± SEM of independent experiments as indicated (n).

Surprisingly we noticed that while more than 80% of ASC and dermal fibroblasts were surviving to CAP treatment, they immediately stopped to proliferate compared to untreated cells (Fig. 1B). This effect was maintained over 14 days (Fig. 2A). Cells did not even recover their expansion potential after passaging (Fig. 2B) while cell mortality remained unchanged (data not shown). Consistent with proliferation arrest we showed that cells mainly blocked in G0/G1 6 hours after CAP treatment leading to a significant decrease in the amount of cells in G2/M (Figure C). Using EdU incorporation we observed a 14 to 25% decrease in ASC and dermal fibroblasts in S phase 24 hours after treatment (24.8 ± 0.8% in untreated versus 10.8 ± 3.7% in treated ASC; and 37.3 ± 5.9% in untreated versus 11.6 ± 1.7% in treated dermal fibroblasts, Fig. 2D).

**Figure 3:**
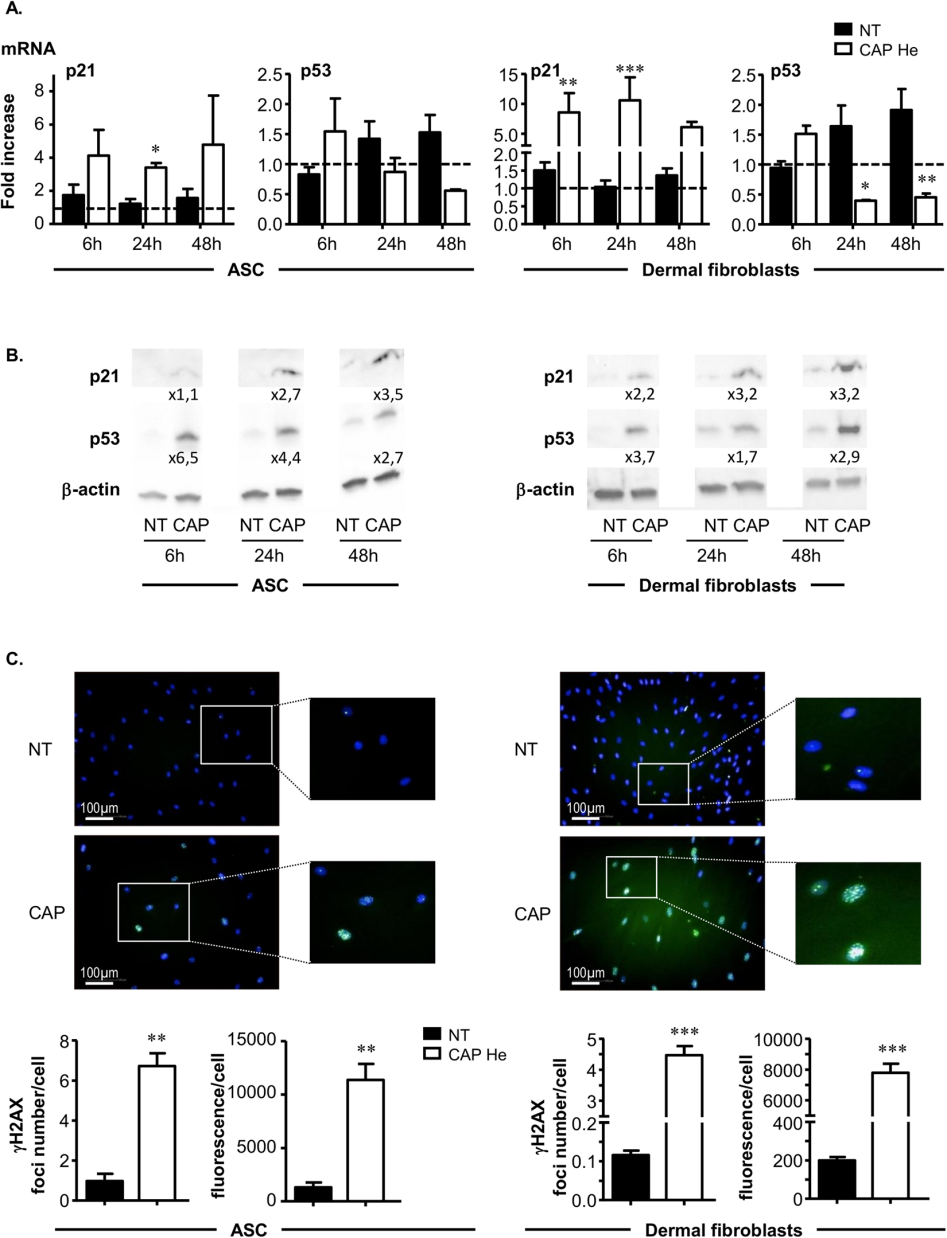
CAP He treatment is associated with an increase in p21 p53 and DNA damage. (**A**) ASC and dermal fibroblasts were treated (CAP or NT) and p21 and p53 mRNA expression was quantified by RT-qPCR at indicated times. Results are presented in fold increase relative to their basal expression at day 0 (dot line). **(B)** p21 and p53 protein level in treated cells was assessed by Western-blot. Quantification corresponds to the fold increase in band intensity of CAP-treated cells normalized to corresponding untreated cells (NT) and divided by β-actin signal. (ASC, n = 3 to 7; fibroblasts, n = 4 to 9). **(C)** Cells were treated and fixed 7 days after treatment to evaluate DNA damage foci by γ-H2AX immunofluorescence. Images were acquired using the high content imaging system Operetta (Perkin Elmer, lens x20) to quantify immunofluorescence and foci number (n = 3). Data represent mean ± SEM of independent experiments as indicated (n).

This observation was consistent with the expression level of transcription factors involved in cell cycle regulation. The cyclin-dependent kinase inhibitor p21 is known to inhibit the activity of cyclin CDK2, CDK1 and CDK4/6 complexes, leading to cell cycle arrest between G1 and S phase. We observed a 4 fold increase for ASC and 6 to 10 fold increase for dermal fibroblasts in p21 mRNA level after CAP treatment (Fig. 3A). This increase in p21 gene expression was associated with a 3 fold increase at the protein level observed after 48 hours (Fig. 3B). In addition we studied the p53 transcription factor, a stress sensor activated to maintain genome integrity by regulating the expression of target genes involved in cell cycle arrest such as p21. p53 mRNA content tended to decrease after CAP treatment (Fig. 3A), whereas the p53 protein level was clearly increased up to 6.5 fold for ASC and 3.7 fold for dermal fibroblasts as early as 6 hours after CAP treatment (Fig. 3B). It is reported that the protein p53 is stabilized and activated in response to stress such as DNA damage and hypoxia, suggesting that efficient p53 regulation rather occurs at post-transcriptional level in accordance with our results^31^. Taken together our data show that after CAP He treatment, ASC and dermal fibroblasts stop their normal proliferation and come into a quiescent state, without massive cell death. The cell cycle arrest is associated with a rapid increase in p53 and p21 content and seems to be irreversible, as cells never recover their proliferation rate on long term culture or after passaging.

### Irreversible exit from cell cycle is associated with DNA damage

Cell cycle arrest and p53 accumulation may occur in response to genotoxic stress. We thus looked for DNA damage in CAP treated cells by detecting phosphorylated histone H2AX (γ-H2AX) foci formation. We observed that 7 days after CAP exposure both treated cells showed an increase in γ-H2AX accumulation in their nuclei (Fig. 3C). Quantification revealed a high foci number per nucleus in treated cells; 6.7 ± 0.49 and 4.4 ± 0.2 for treated ASC and dermal fibroblasts respectively compared to 0.9 ± 0.2 and 0.1 ± 0.008 in non-treated cells. These γ-H2AX foci number increases correlated with a strong increase of γ-H2AX foci fluorescence intensity. Results thus demonstrate that CAP He treatment induces DNA damages in human ASC and dermal fibroblasts, such as double stranded breaks identified by γ-H2AX foci known to be recruited at damaged sites.

### ASC and dermal fibroblasts acquire some senescent characteristics after CAP He treatment

**Figure 4:**
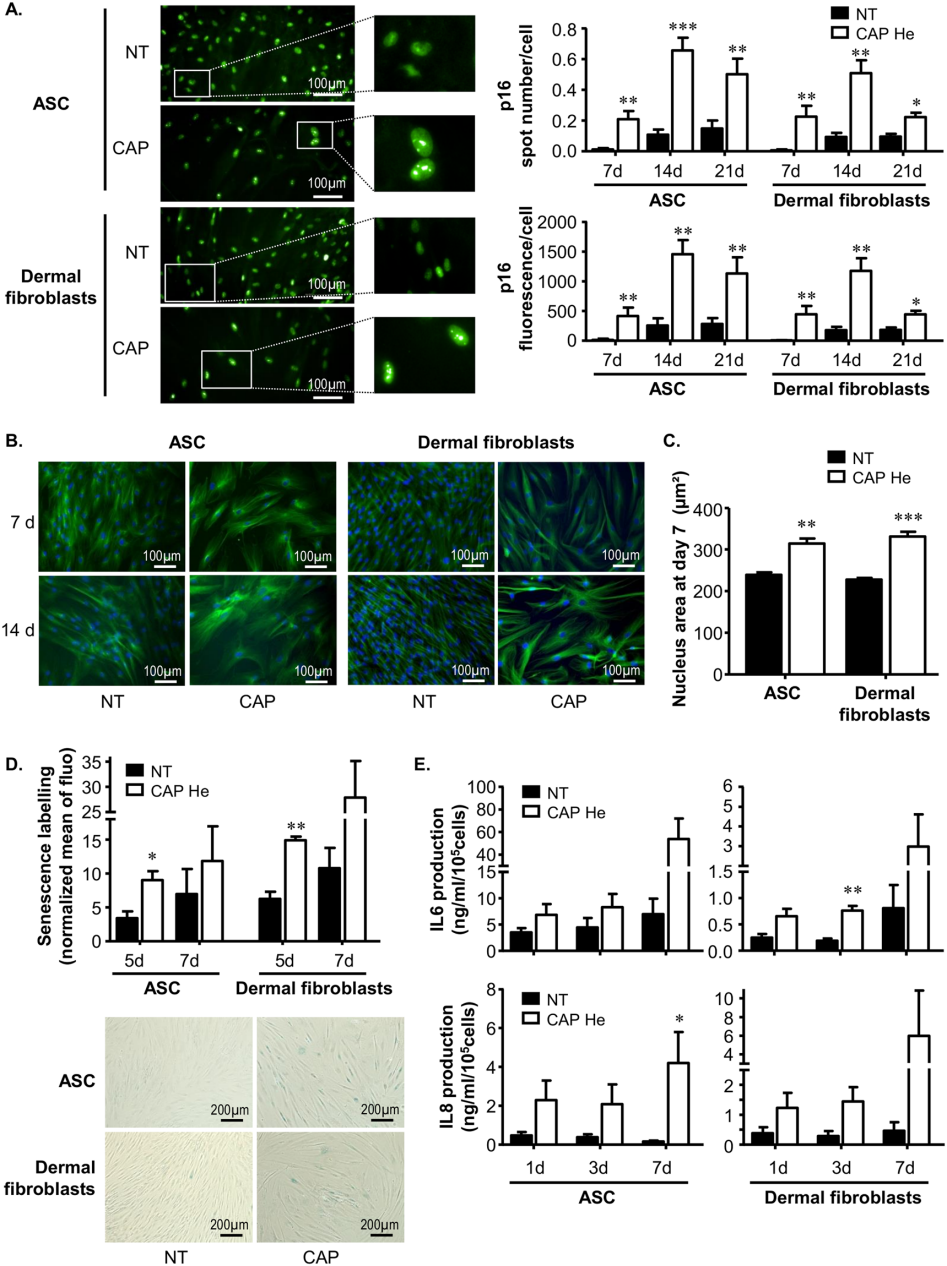
Acquisition of a senescent phenotype by CAP He treated ASC and dermal fibroblatsts. (**A**) ASC and dermal fibroblasts were treated (CAP or NT) and nuclear p16 was imaged by immunofluorescence. Images were acquired using the high content imaging system Operetta (Perkin Elmer, lens x20) to quantify p16 nuclear spots number per cell and fluorescence intensity per cell (means of 5 fields). (**B**) Senescence associated-morphological change was observed 7 and 14 days after treatment. Cells morphology was evaluated by DAPI (nuclear) and β-tubulin (cytoskeleton) labeling using the high content imaging system Operetta (Perkin Elmer, lens x20). Pictures were taken with a Nikon, Eclipse, TE2000-S microscope (x200 magnification) (n = 4). **(C)** Nuclear size was additionally quantified (n = 4). (**D**) Senescence associated-β-galactosidase (SA-β-gal) was quantified 5 and 7 days after treatment by flow cytometry with a substrate (C12-FDG). Quantification is represented by the Mean of Fluorescence Intensity (MFI) normalized to the same condition without labelling. SA-β-gal was also evaluated by biochemical detection (blue staining) and pictures were taken at day 7 with a Nikon, Eclipse, TE2000-S microscope (ASC, n = 3 to 4; fibroblast, n = 3 to 5). **(E)** Senescence associated cytokine secretion was evaluated in treated cells supernatant for IL-6 and IL-8 by ELISA (n = 3 to 6). Data represent mean ± SEM of independent experiments as indicated (n).

Consistent with resistance to apoptosis, cell cycle arrest and p53 stabilization we wonder if cells were entering into senescence. Cellular senescence can be driven by two main pathways to stop cell proliferation; the p53-p21 pathway and the p16-pRB pathway. The cyclin-dependent kinase inhibitors p21 and p16 inhibit the retinoblastoma protein (RB) phosphorylation thus preventing its inactivation. Active RB can then blocks cell cycle progression and restrains cell growth. The expression of p16 is thus considered as a main senescence-associated transcription factor, and was evaluated in CAP treated cells. Interestingly we observed that nuclear p16 expression was gathered into nuclear spots. Quantification of these spots showed that CAP treated ASC and dermal fibroblasts had a strong increase in p16 spots number per cell, consistent with a strong increase in p16 spot fluorescence per cell at 7, 14 and 21 days after CAP treatment (Fig. 4A).

In addition we looked for characteristic morphological changes described with cell undergoing senescence^32^. CAP treated cells appeared enlarged and flattened. Cytoplasmic β-tubulin labeling showed a spread shape and long membrane extensions in treated cells (Fig. 4B). Quantification of nucleus area confirmed that CAP treated cells had bigger nuclei than non-treated cells (Fig. 4C). Then, we studied the lysosomal Senescence-Associated β-Galactosidase (SA-β-Gal) known to be overexpressed in senescent cells. Biochemical detection of SA-β-Gal showed an increase of the specific blue staining 7 days after CAP treatment supporting that CAP treated cells underwent senescence (Fig. 4D). Accordingly similar conclusions were reached using a quantitative technique to evaluate SA-β-Gal by flow cytometry with C_12_FDG (5-Dodecanoylaminofluorescein Di-β-D-Galactopyranoside), a substrate of the enzyme becoming fluorescent when cleaved that significantly increased in CAP treated cells at 5 days after treatment and maintained at 7 days (Fig. 4D). Finally an important feature of senescent cells is to have a specific secretome named SASP. This SASP is complex, cell type dependent and includes pro-inflammatory cytokines secretion. We tested CAP treated cells for the two major pro-inflammatory cytokines IL-6 and IL-8 reported to be SASP-associated^33^. We observed in the supernatant of CAP treated ASC and dermal fibroblasts a clear increase in the secretion of IL-6 and IL-8 starting from day 1 after treatment and maintained over time (Fig. 4E). All together these data are in accordance with cellular senescence showing that human primary ASC and dermal fibroblasts may resist and survive to CAP He exposure to rapidly enter into senescence.

### CAP He treatment induces a metabolic switch in ASC and dermal fibroblasts

**Figure 5:**
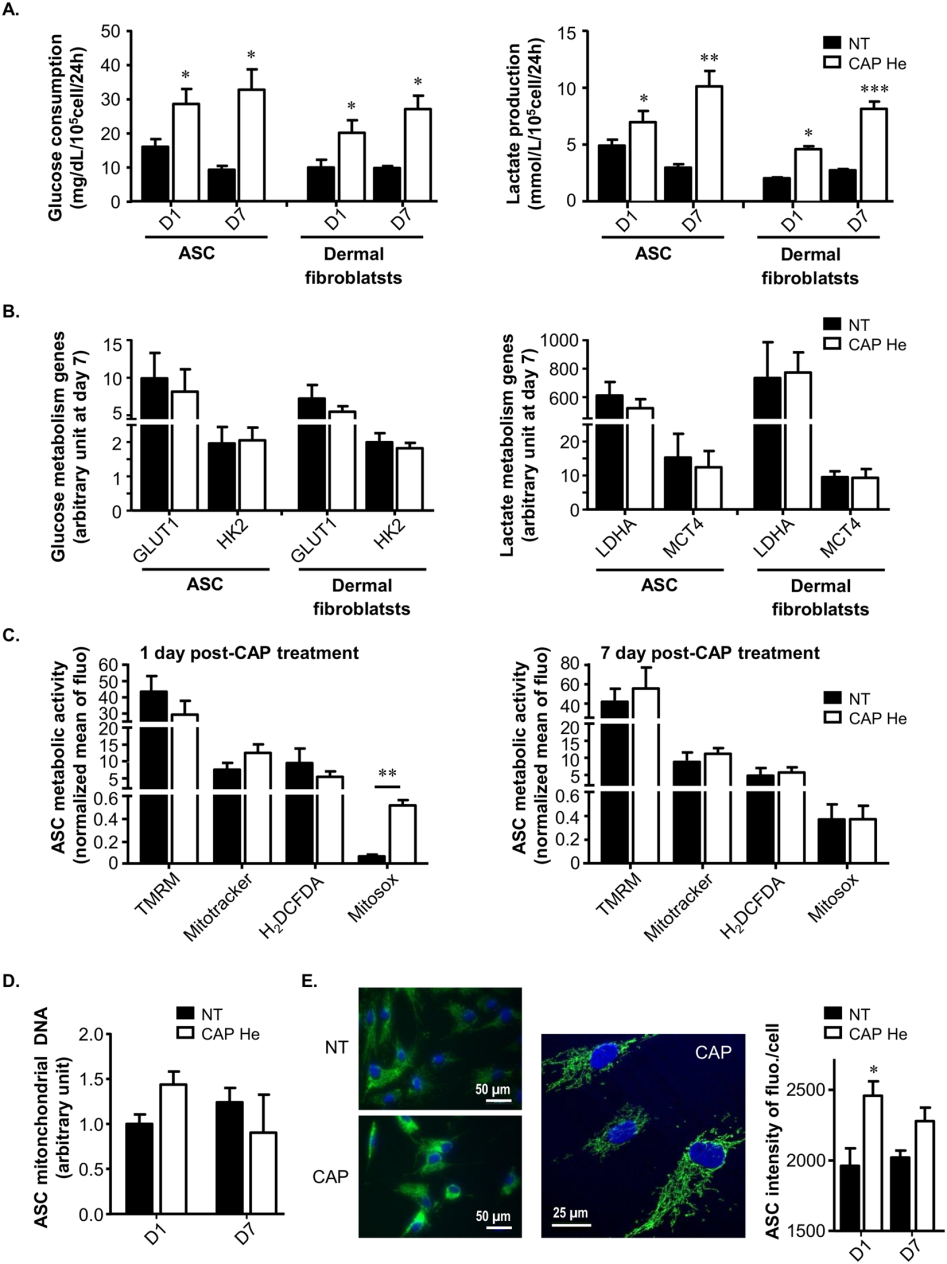
The senescent phenotype is associated with a switch to a glycolytic metabolism and mitochondria modifications. (**A**) ASC and dermal fibroblasts were treated (CAP or NT) and glucose and lactate concentrations were measured in supernatant 1 (D1) and 7 (D7) days after the treatment (n = 3 to 6). **(B)** The level of GLUT1, HKII, LDHA and MCT4 mRNA expression was quantified by RT-qPCR 7 days after the treatment (ASC n = 6; fibroblasts n = 3). **(C)** ASC were treated (CAP or NT) and tested after 1 and 7 days for their mitochondrial membrane potential using the TMRM probe, the mitochondria mass using the MitoTracker probe, intracellular ROS production using H_2_DCFD and mitochondrial ROS production using Mitosox probe. Quantification is represented by the Mean of Fluorescence Intensity (MFI) normalized to the same condition without labelling (n = 4). **(D)** ASC were treated (CAP or NT) and tested for mitochondrial DNA quantification 1 and 7 days after treatment (n = 3). **(E)** ASC were treated (CAP or NT) and stained by immunofluorescence for mitochondria content 1 and 7 days after treatment. Pictures were taken with a Nikon, Eclipse, TE2000-S microscope (x400 magnification) with the same calibration and a zoom was realized with a confocal LSM 780 (Zeiss) (x630 magnification). Quantification is performed using the high content imaging system Operetta (Perkin Elmer, lens x20) (n = 3). Data represent mean ± SEM of independent experiments as indicated (n).

Senescent cells can maintain a high glycolytic metabolic rate despite their non-dividing state that is associated with altered mitochondrial function^34^. Such metabolic changes are crucial for the induction and maintenance of senescence. We thus tested if CAP treated ASC and dermal fibroblasts operate such metabolic switch. For this investigation, glucose and lactate were measured in the culture medium, allowing to calculating glucose consumption and lactate production normalized to cell number during 24 hours (Fig. 5A). We observed that CAP treated cells had an increase in glucose consumption and lactate production from the first day after treatment that was maintained over time. Gene expression was evaluated 7 days after treatment and showed that CAP treated cells showing no significant difference in genes related to glucose metabolism (Fig. 5B) including the glucose transporter GLUT1 and the hexokinase-2 (HK2) which phosphorylates glucose during the first step, as well as genes related to lactate metabolism (Fig. 5B) with the lactate dehydrogenase-A (LDHA) which converts pyruvate into lactate and the monocarboxylate transporter-4 (MCT4) which exports lactate produced from glycolysis. Results lead to the conclusion that CAP treatment induces a metabolic adaptation in favor of a glycolytic metabolism whereas cells stop their proliferation. As the switch in cell metabolism can be linked to mitochondria, we then evaluated mitochondrial activity and integrity. The mitochondrial membrane potential was tested using the TMRM probe (Tetramethylrhodamine methyl ester) that tended to decrease of mitochondrial membrane potential one day after CAP treatment (Fig. 5C), possibly resulting from CAP electromagnetic field^28^. Mitochondria mass evaluation was performed using the MitoTracker probe, highlighting an increase at 1 day after CAP treatment (Fig. 5C). The same tendency was found when assessing the mitochondrial DNA content (Fig. 5D) and the mitochondrial immunofluorescence intensity (Fig. 5E) in cells one day after treatment. Finally intracellular ROS production was measured using H_2_DCFDA (2′,7′-dichlorodihydrofluorescein diacetate) and did not show any significant changes 1 or 7 days after CAP treatment (Fig. 5C), even though an increase was observed during the first 2 hours following treatment (data not shown). Comparatively, mitochondrial ROS production measured using the Mitosox probe showed a high increase at day 1 after treatment, that return to basal level at day 7 (Fig. 5C). All together these data suggest that cells react to this CAP-generated stress by rapidly increasing their mitochondrial content and changing their metabolism to glycolysis, consistently with the senescence phenotype^34^.

### Modifications in ASC and dermal fibroblasts intrinsic properties after CAP He treatment

**Figure 6:**
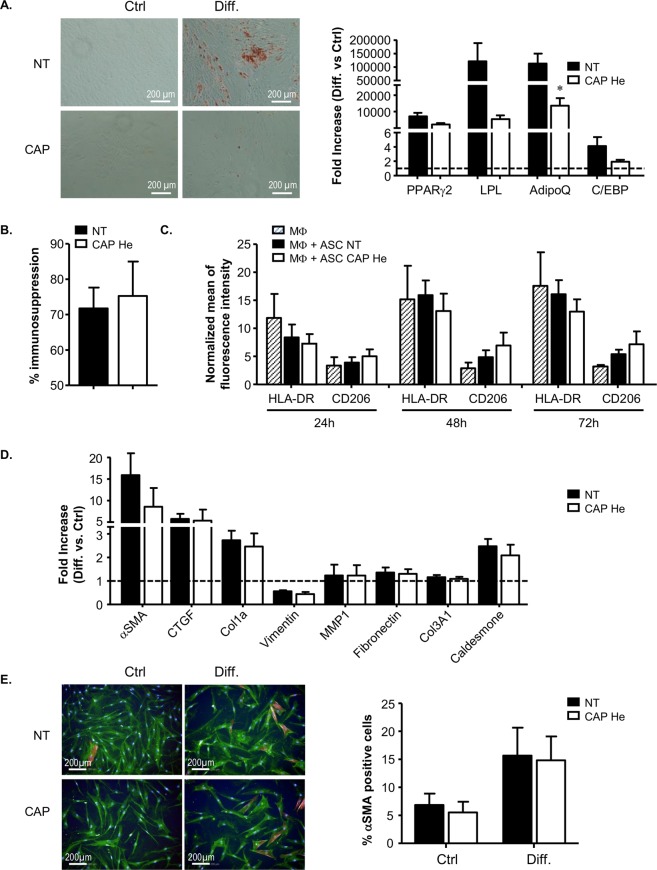
Analysis of senescent ASC and dermal fibroblasts functional properties. (**A**) ASC adipogenic potential was assessed 7 days after ASC treatment (CAP or NT) by induction of differentiation (Diff.) and analysis by Oil-Red-O staining of lipid droplets (day 21 after induction of differentiation) and by RT-qPCR for adipogenic gene expression PPARγ2, LPL, Adiponectin and C/EBPα (day 14 after induction of differentiation) in comparison to ASC cultivated in control medium (Ctrl, no differentiation). Pictures were made with Nikon, Eclipse, TE2000-S microscope. RT-qPCR results are represented in fold increase to control medium condition (dot line) for the different genes (n = 4). **(B)** ASC were tested for their immunosuppression activity by co-cultivating ASC with activated T lymphocytes (TL) labelled with the CFSE marker. The percentage of immunosuppression is determined by flow cytometry after 5 days (proliferating TL = % TL CFSE negative) (n = 6). **(C)** Effect on macrophage polarization was performed by co-cultivating ASC with M0 macrophages (monocytes cultivated 6 days with M-CSF) and macrophage phenotype analysis by flow cytometry at indicated times for membrane markers CD45, HLA-DR and CD206. Data are represented in mean of fluorescent intensity (MFI) normalized to isotype control among living macrophages (DAPI negative and CD45 positive cells) (n = 4). **(D)** Dermal fibroblasts 7 days after treatment (CAP or NT) were tested for their differentiation into myofibroblast under TGF-β stimulation by RT-qPCR for αSMA, CTGF, vimentin, MMP1, fibronectin, collagen COL1a, COL3A1 and caldesmone (72 hours). Data are represented in fold increase to control media (n = 3). **(E)** Dermal fibroblast activation was also estimated by α-SMA labeling and imaging using the high content imaging system Operetta (Perkin Elmer, lens x20) (n = 4). Data represent mean ± SEM of independent experiments as indicated (n).

Finally we evaluated if senescence in ASC and dermal fibroblasts was associated with changes in their intrinsic properties. ASC were challenged for their ability to differentiate into adipocytes and their immunomodulatory potential. 7 days after CAP treatment, adipocyte induction was performed in differentiation medium or not (control medium) for 14 days. We observed that senescent ASC can differentiate into adipocyte but to a much lower extent than non-treated cells as shown by adipogenic genes expression evaluation (PPARγ2, LPL, Adiponectin and C/EBPα) and lipid droplets accumulation (Fig. 6A). Immunosuppressive property on T lymphocyte proliferation was tested. We observed that control ASC induced a decrease of T lymphocyte proliferation as described^35^ and this percentage of immunosuppression was not affected in senescent CAP-treated ASC (Fig. 6B). Finally, we evaluated the capacity of senescent ASC to trigger macrophage polarization based on the membrane expression of HLA-DR, a marker of M1 pro-inflammatory macrophages and CD206, a marker of M2 anti-inflammatory macrophages^36,37^. In co-culture with non-treated ASC, M0 macrophages tended to decrease HLA-DR while increasing CD206. This effect was even more effective with senescent ASC (Fig. 6C). All together CAP-induced senescent ASC are less effective to differentiate into adipocyte, maintain their immunosuppression potential and tend to support M2 polarization of macrophages.

Dermal fibroblasts were tested for their ability to differentiate into myofibroblasts involved in wound closure by contraction. 7 days after CAP treatment, myofibroblast induction was stimulated by TGF-β. No remarkable modification in myofibroblastic genes expression relative to the α-SMA, fibrogenic factor CTGF, to the extracellular matrix protein collagen, vimentin, fibronectin, and caldesmone involved in contractile activity, were observed in senescent fibroblasts (Fig. 6D). Moreover, no modification in α-SMA protein expression was observed by immunofluorescence protein quantification (Fig. 6E). Together, these data reveal no major modifications in myofibroblast differentiation capacity of CAP-induced senescent fibroblasts.

Taken together this study shows that primary human ASC and dermal fibroblasts can survive to CAP He exposure but display deleterious effects such as DNA damage and mitochondrial membrane potential disruption. Cells react by definitively stopping proliferation, acquiring all the features of senescent cells, switching for a glycolytic metabolism to survive while maintaining their specific biological functions.

## Discussion

In this study we showed that, after CAP He exposure *in vitro*, healthy human skin-derived primary cells resist to apoptosis induction and undergo cellular senescence. To our knowledge this is the first study reporting such effects on human cells isolated from skin samples in primary culture. CAP-induced senescence has only been described on malignant melanoma cell lines treated with a distinct plasma device producing plasma in air^38,39^. It was initially proposed that CAP treatment could be used as a potent anti-tumor strategy by targeting skin cancer cells to selectively induce apoptosis^40,41^. Recently, moderate CAP production opened new perspectives as an alternative approach to selectively induce senescence in melanoma to stop tumor growth^38,39^. With the present study we now show that healthy skin cells sensitivity to CAP has also to be considered, rising the control of CAP parameters and its diffusion into skin layers as the determinant point to monitor to avoid any adverse event on healthy tissue.

Among the various human primary cell types tested in the present study, different sensitivities to apoptosis induction were observed, the most sensitive cells being T lymphocytes and to a lesser extend macrophages as previously described^42,43^. It is however reported that in a lower dose-dependent manner CAP treatment may rather activate immune cells^44^. Thus selective impact of CAP on immune cells remains to be further investigated regarding to the major contribution of immune cells in the wound healing process. Conversely our results show that skin cells such as keratinocytes, dermal fibroblasts and ASC are greatly resisting to CAP-induced cell death but unexpectedly entered into senescence. Effect of CAP on human keratinocytes, mainly the HaCaT cell line, has been widely explored with results ranging from no effect or stimulation to inhibition of apoptosis, proliferation, differentiation, migration, or antioxidant defenses^19–21,23,25,45,46^. Fibroblasts from different origin (dermal, oral, hepatic, and intestinal) are also reported to behave differently depending on CAP conditions. Cells may enter cell death by necrosis or apoptosis, may proliferate, migrate, change their gene expression program or get activated^18,22,27,47,48^. Comparatively few data are available concerning mesenchymal stromal cells (MSC). CAP treatment was shown to have no effect on human bone marrow MSC in comparison to an induction of apoptosis in sarcoma osteogenic SaOS-2 cells^49^. In fact CAP was mostly used to activate bioengineered scaffold surfaces for a better cell attachment and differentiation for bone and cartilage regeneration^50,51^. Only one study was dedicated to human ASC reporting that CAP treatment promotes proliferation via a NO-dependent pathway without affecting cell viability and adipogenic potential^52^. On the contrary inhibition of adipose differentiation without cytotoxic effect was reported on the murine 3T3-L1 preadipocyte cell line and by decreasing adipose tissue mass in mouse injected with plasma treated medium^53^. Our study brought new insights showing that CAP treatment may also trigger senescence in ASC and dermal fibroblasts. Taken together scattered results are obtained at the cell level, probably due to non-standardized sources, devices and procedures used to generate plasma, leading to different RONS production in quality and quantity. However a constant and robust finding is that protective or beneficial effects are generally obtained with mild CAP whereas cell viability is compromised with strong CAP on non-malignant cells. In studies addressing the mechanism of action, stable RONS production, including hydrogen peroxide, nitrite and nitrate, is generally incriminated as the primary signal initiating those stress-activated cellular pathways.

Human dermal fibroblasts and ASC treated in our CAP conditions present all the features of senescence. Senescent cells are characterized by a set of biomarkers that includes a permanent growth arrest with morphological changes, senescence-associated β-galactosidase activity, tumor suppression network activation involving p53 and p16/Rb pathways, senescence-associated heterochromatin foci, a senescence-associated secretory phenotype (SASP)^54,55^ without telomere shortening mostly observed in the case of replicative senescence. Exogenous oxidative stress may lead to MSC senescence as demonstrated with sub-lethal doses of hydrogen peroxide (100–150 µM H_2_O_2_)^56^. In our conditions CAP treated cells may react by developing rapid anti-oxidant activity to take in charge this overload of ROS, so that the intracellular level of ROS remains unchanged over time. Meanwhile this oxidative stress could result in the observed p53 activation, DNA damage and impairment in mitochondrial function with a transient increase in mitochondrial ROS in accordance with previous observations^57–59^. The transcription factor p53 initially known as a tumor suppressor can in fact trigger opposed decisive roles by promoting cell survival as well as cell death. Under acute stress p53 acts as a determinant factor lowering ROS level to reduce damage and promote survival and repair whereas under more severe or sustained stress p53 makes cell switching to senescent and apoptotic programs to eliminate altered cells^60–62^. This is in line with our findings where CAP-generated stress rapidly activates p53 and stop proliferation (6 hours). Cells manage intracellular ROS level and augment glycolytic metabolism but fail to prevent DNA damage accumulation driving the cells to senescence within few days. This glycolytic switch in the presence of oxygen may be a way to preserve cell integrity by feeding the pentose phosphate pathway and providing NADPH to favor antioxidant defenses, as reported in irradiated-senescent human fibroblasts^63^.

Besides ROS production mitochondria dysfunction may be linked to cellular senescence through many signaling pathways such as mitochondrial mass and dynamic, redox state, electron transport chain, energetic balance, metabolic flux and calcium homeostasis^64^. Weather mitochondrial dysfunction is a consequence or has a causal effect in senescence is still questioned. However a recent study reports that mitochondria depletion in fibroblasts is sufficient to reduce cellular senescence features induced by oxidative stress, oncogene activation or replicative exhaustion^65^. Mitochondria are thus required for senescent fate-determination and could be targeted in anti-aging strategy for example. In agreement with other models of senescence^66,67^ we found that mitochondria is a key element; together with the senescent phenotype cells respond to CAP exposure by increasing mitochondrial mass and redirecting their metabolism to glycolytic flux thus limiting respiratory chain function and ROS production.

In our experiments CAP treated cells maintained their *in vitro* functional properties. Their differentiation potential is preserved even though reduced for ASC differentiation into adipocyte. We showed that stress-induced senescence did not alter ASC immunomodulation potential. Comparatively it was reported that replicative senescence caused a decline in ASC efficiency to inhibit T lymphocyte proliferation^35^. However the same study also demonstrated that this potential could be restored under strong inflammatory stimulation. In line with our findings this suggests that the immunomodulation property is not loss with senescence.

Cellular senescence is implicated in some pathological disorders as well as physiological process. Long term accumulation of senescent cells is incriminated in tissue dysfunction and disruption like in chronic disease or aging, whereas a transient presence may rather promote tissue regeneration^68,69^. During development programmed-senescence contributes to morphogenesis and organogenesis by removing unwanted cells. After injury senescence participates to tissue remodeling through a SASP-dependent immune cells recruitment to eliminate injured cells. Additionally an immune-mediated clearance of senescent cells contributes to limiting proliferation and fibrosis during tissue reconstruction^68,69^. Whether the presence of CAP-induced senescent cells would be beneficial or detrimental in the wound healing process remains to be firmly determined *in vivo*. Investigations in wound healing models are now required to assess whether such senescent cells may favor skin regeneration or accumulate and compromise tissue healing. Nevertheless local and short exposure to CAP in order to provoke transient senescence to favoring tissue regeneration could be considered in complicated or compromised skin wound healing.

## Conclusion

We demonstrate that human skin fibroblasts and ASC are resisting to death and apoptosis, stop their proliferation in culture to rapidly undergo into senescence after CAP exposure while maintaining and adapting their functional properties. They expressed a senescence-associated secretory phenotype (SASP), and exerted slightly modified functional properties in term of secretion, immunomodulation and differentiation potential, suggesting that dermal fibroblasts and ASC may be targeted through a CAP treatment to significantly influence the outcome of the healing process. Localized and controlled CAP exposure may be a promising approach to avoid wound healing impairment ranging from persistent open wound to excessive, disorganized hypertrophic or keloid scaring by targeting ASC and dermal fibroblasts biological properties.

## Methods

### Helium cold atmospheric plasma (CAP He) device

The plasma process was composed of the reactor, a dielectric tube in alumina in which a tungsten filament was inserted and a high-voltage was powered. A metallic cylinder fixed around the dielectric tube and grounded allowed the application of high electric fields between the tungsten filament and the cylinder. This DBD configuration allowed limiting the current and avoided the formation of an electrical arc. In order to control the gaseous environment of ionization waves, a quartz tube was placed around the DBD-based experimental device. The plasma process consisted in the production of guided ionization waves at atmospheric pressure and room temperature as previously described^70^. Process gas was Helium at a 1.7 standard liters per minute (slm) flow rate. Plasma was generated by applying a 7.5 kV, 10 kHz, 1% duty cycle, with a positive nanosecond pulsed wave potential between the two electrodes.

### Human blood cells isolation

Human buffy coat samples were provided by the French blood bank (Etablissement Français du Sang Pyrénées-Méditerranée) to collect mononuclear cells (PBMC) on density gradient with Ficoll (Eurobio) and then isolate by depletion monocytes (human monocyte isolation kit, Milteny) or T lymphocytes (human Pan T cell isolation kit, Milteny) with the autoMACS (Milteny Biotec). Macrophages M0 were obtained by cultivating monocytes 6 days with 100 ng/ml human M-CSF (Peprotech). M0 macrophages were cultivated during 24 h with 1 µg/ml LPS (InvivoGen) and 20 ng/ml IFN-γ (Peprotech) for M1 polarization or with 20 ng/ml IL-4 (Peprotech) for M2 polarization.

### Human tissue cell isolation

Adult skin tissue samples were obtained from donors undergoing elective abdominal dermolipectomy (age 25–58 years, BMI <30, n = 77) with no objection certificate according to the bioethic law no. 2004–800 of August 6, 2004. Keratinocytes were isolated after epidermis and dermis digestion with 10 mg/ml dispase solution (Sigma Aldrich) in Hank’s Balanced Salt Solution (HBSS, ThermoFisher) then trypsine solution (Life technologies) to dissociate epidermis after filtration (100 µm, EMD Millipore). Keratinocytes were cultivated in Dermalife K medium (Cell System) supplemented with 1% ASP. Skin fibroblasts were obtained using dermis explants cultivated in α-MEM medium (Life technologies) with 1% ASP and 20% FBS. Adipose Stromal Cells (ASC) were obtained by digestion of subcutaneous adipose tissue with 0,4 U/ml collagenase NB4 (SERVA electrophoresis) solution in α-MEM medium then removal of floating adipocytes to isolate and culture the stromal vascular fraction in α-MEM medium with 1% ASP and 10% FBS. Cells were used from passage 0 to 4 (P0 to P4).

### CAP He treatment

Cells were seeded in 6 wells plates 7 days before CAP treatment for macrophages (1 × 10^6^ cells/well) or 1 day before CAP treatment for T lymphocytes (2 × 10^6^ cells/well), keratinocytes (0,5 × 10^6^ cells/well), ASC and dermal fibroblasts. Dermal fibroblasts and ASC (control and treated conditions) were seeded at 1 × 10^5^ cells/well. For experiment related to senescence (Figs 3C, 4), metabolic (Fig. 5) and functional (Fig. 6) studies non-treated ASC and dermal fibroblasts had to be seeded at 2 × 10^4^ cells/well for long-term culture (over 5 days) to maintain of their proliferating rate and metabolic activity over time. Indeed, non-treated cells seeded at 1 × 10^5^ cells/well reached confluence around day 5 (Fig. 2A) and stopped proliferation with a reduced metabolic activity due to contact inhibition which may interfere with CAP-induced effects in treated cells for results analysis. All cells were treated for 3 min in 2 ml PBS and incubated 1 h at 37 °C. PBS was then replaced by fresh medium. CAP treated cells were compared to control cells non-exposed to plasma (non-treated, NT).

### Living/apoptotic/dead cell ratio analysis

Apoptosis and necrosis were evaluated with the AnnexinV-APC/PI labeling kit (Apoptosis detection kit eBioScience) by flow cytometry with the LSR Fortessa system (B&D) and analyzed with the Kaluza software (Beckman). Living cells were AnnexinV/PI negative, apototic cells were AnnexinV positive and dead cells were PI positive.

### Cell numeration

Trypsinized cells (Life technologies) were counted on Malassez slide after Trypan blue dilution 1:2 (Life technologies). Results were expressed as cell number per cm².

### Cell cycle analysis by EdU incorporation and flow cytometry

Cell cycle was studied using the Click-iT Plus EdU Pacific Blue Flow Cytometry Assay Kit (ThermoFisher) and EdU staining was analyzed by flow cytometry (LSR Fortessa, B&D).

### RT-qPCR and mitochondrial DNA quantification

RNA extraction was performed according to manufacturer recommendations (RNeasy Micro Kit, Qiagen) to perform Reverse Transcription (RT) with high-capacity cDNA reverse transcription kit (TerhmoFisher) on ProFlex thermal cycler (Life technologies). Total DNA was extracted from ASC with AllPrep DNA/RNA Mini kit (Qiagen). Mitochondrial DNA was quantified using mitochondrial gene MT-ND1 (NADH-ubiquinone oxidoreductase chain 1). Quantitative polymerase chain reactions (qPCR) was performed with 2 µL of cDNA or DNA, 1.5 µL forward and reverse primers at 2 µM and 5 µL of fast SYBR green master mix (ThermoFisher). PCR cycles were performed in StepOne (ThermoFisher) with PUM or β-actin (genomic DNA gene to normalized mitochondrial DNA quantification) as the housekeeping gene. Analysis was made with the StepOne software, with cycle threshold evaluation (CT). Mean of duplicates CT was used to calculate deltaCT = CT_gene_ − CT_PUM/b-actine_. Data were analyzed in 2^−deltaCT^ or in fold increase of 2^-deltaCT^ to control cells. Primer sequences are listed in Supplementary Table 2.

### Western blot

Proteins were extracted from frozen cell pellets with RIPA solution containing 150 mM NaCl, 50 mM TRIS HCl pH 7.4, 0.1% SDS, 0.5% deoxycholate, 1% NP40, 1 mM PMSF, 1X Complete Mini, 1 mM sodium orthovanadate (BioRad) and dosed with the Micro BCA Protein Assay Kit (Thermo Scientific). 30 µg proteins were separated on 4–20% Mini-PROTEAN TGX precast protein Gel (Bio-Rad) then transferred on a PVDF membrane with the Trans-Blot Turbo Mini PVDF Transfer (Bio-Rad). Western blotting was performed using primary mouse anti-human antibodies anti-p21 (1:500, Santa Cruz), anti-p53 (1:500, Santa Cruz) and anti-β-actin (1:10000, BioRad). Secondary goat anti-mouse IgG-HRP conjugate antibody (Bio-Rad) was used at 1:1000 for p21 and p53 detection and at 1:10000 for β-actin detection. Revelation was made with Clarity western ECL kit (BioRad). Reading and pictures were performed with ChemiDoc XRS + system (BioRad) and band quantification was made with the Image Lab software.

### Adipogenic differentiation

7 days after CAP treatment, ASC were placed in differentiation medium (α-MEM, 1% ASP, 10% FBS, 1 µM dexamethasone, 60 µM indomethacine, 500 µM IBMX) (Sigma Aldrich) or control medium (αMEM, 1% ASP, 10% FBS) for 7, 14 or 21 days. RT-qPCR was performed for the peroxisome proliferator-activated receptor (PPARγ2), lipoproteine lipase (LPL), Adiponectin and CCAAT/enhancer binding protein alpha (C/ΕΒΠα) gene expression. Triglycerides were stained with Oil red-O (Sigma-Aldrich). Pictures were taken with a Nikon, Eclipse, TE2000-S microscope.

### Myofibroblastic differentiation

7 days after CAP treatment, fibroblast differentiation into myofibroblast was induced with 10 ng/ml TGF-β1 (Miltenyi) during 72 h. RT-qPCR was performed for the smooth muscle actin (α-SMA), connective tissue growth factor (CTGF), collagen type 1a (Col1a) and 3A1 (Col3A1), vimentin, matrix metalloproteinase 1 (MMP1), fibronectin and caldesmone gene expression. Cells were also labeled with 10 µg/ml WGA-Al488 (ThermoFisher) then with mouse anti-human α-SMA antibody (1:100, DAKO) and finally goat anti-mouse Al594 secondary antibody (1:250, ThermoFisher). Nuclei were stained with DAPI (1:10000, Sigma Aldrich).

### T lymphocytes immunosuppression

7 days after CAP treatment, ASC were plated at 100 000 cells/well before adding beads-activated T lymphocytes (Dynabeads human T activator CD3/CD28, Gibco) labeled with 2.5 µM CFSE (CellTrace CFSE Cell Proliferation Kit, Invitrogen). T lymphocytes quantification was performed after labeling with CD3-APCVio770 (Milteny) and CD45-VioBlue (Milteny) or their respective isotypes by flow cytometry (LSR Fortessa, BD). The % of proliferating T lymphocytes corresponded to living CD45^+^ CD3^+^ cells with decreasing CFSE-FITC labeling with cell division. The % of immunosuppression was calculated: (1-(% proliferating TL with ASC)/(% proliferating TL alone))*100.

### Macrophages/ASC co-culture and phenotyping

7 days after CAP treatment, ASC were co-cultured with M0 macrophages. After 24, 48 or 72 h macrophages were harvested and labeled with CD45-APCVio770 (Milteny), CD206-PE (B&D) and HLA-DR-FITC (B&D) or their respective isotypes before flow cytometry analysis (LSR Fortessa, B&D). Mean fluorescent intensity (MFI) normalized isotype MFI were evaluated on living CD45 positive cells.

### Immunofluorescence

For morphological properties of ASC and dermal fibroblasts, a double staining was performed using rabbit anti-human β-tubulin antibody (1:400, Abcam) then goat anti-rabbit Al488 secondary antibody (1:500, ThermoFisher), and nuclei stained with DAPI. Results were expressed as nuclei size and photos provided for cytoskeleton organization.

For γ-H2AX DNA damages foci, cells were labelled with mouse anti-human phospho-histone H2AX (Ser139) antibody (1:500, Merk Millipore) then donkey anti-mouse Al488 secondary antibody (1:200, ThermoFisher) and nuclei stained with DAPI. γ-H2AX foci number per cell and fluorescence intensity per cell were calculated.

Cells were labelled with rabbit anti-human p16 antibody (1:270, Abcam) then goat anti-rabbit Al488 secondary antibody (1:200, ThermoFisher) and nuclei stained with DAPI. Results were expressed as p16 nuclear spots number and fluorescence intensity per cell.

Mitochondria were labelled with mouse anti-human mitochondrial Ab2 MTC02 (1:200, NeoMarkers) then donkey anti-mouse Al488 secondary antibody (1:400, ThermoFisher) and DAPI. Al488 fluorescence intensity per cell was calculated.

Images were taken with the high content imaging system Operetta (Perkin Elmer).

### Glucose and lactate measurement

7 days after CAP treatment, glucose and lactate were measured in cell supernatants with Contour XT (Bayer) and Lactate Pro 2 (Arkray) kits respectively.

### Metabolic parameters evaluation by flow cytometry

1 and 7 days after CAP treatment, ASC were harvested and incubated or not (negative control) with metabolic probes: 100 nM TMRM, 75 µM 2DOG, 2 µM H_2_DCFDA, 150 nM MitoTracker™ Green FM and 500 µM Mitosox (ThermoFisher) before evaluation by flow cytometry (LSR Fortessa, BD). Mean of fluorescence intensity (MFI) of markers and normalized MFI (substracted by negative controls MFI) were evaluated on living cells (analysis on Kaluza software).

### Senescence-Associated β-Galactosidase (SA-β-Gal) histochemical staining

7 days after CAP treatment, cells were fixed and stained for SA-β-Gal according to manufacturer recommendations (Senescence Detection Kit, BioVision). Pictures were taken with a Nikon, Eclipse, TE2000-S microscope.

### Senescence-Associated β-Galactosidase (SA-β-Gal) detection by flow cytometry

5 or 7 days after CAP treatment cells were incubated or not (negative control) with 33 µM of 5-Dodecanoylaminofluorescein Di-β-D-Galactopyranoside substrate (C12FDG, ThermoFisher) and the uptake was quantified by flow cytometry (LSR Fortessa, B&D) on FITC channel. Mean fluorescent intensity (MFI) normalized to isotype MFI were evaluated on living cells (analysis on Kaluza software).

### Enzyme-Linked Immuno-Sorbent assay (ELISA)

Supernatant from cell culture was recovered at 1, 3 and 7 days after treatment and cytokines were measured according the human IL-6 and IL-8 Standard ABTS ELISA Development Kits (PeproTech). Optical density was measured at 405 nm with Varioskan (ThermoFisher).

### Statistics

Histograms represent mean ± SEM of independent experiments (n). A two way ANOVA with Boneferroni correction was use for time course experiments and parametric unpaired t-test was used to compare single point data analysis from non-treated (NT) versus treated (CAP) cells originating from a same donor. *p < 0.05; **p < 0.01; ***p < 0.001.

## Supplementary information

Supplemenrary information
