# Supplementary material for: Short exposure to cold atmospheric plasma induces senescence in human skin fibroblasts and adipose mesenchymal stromal cells
Source: Sci Rep. 2019 Jun 17;9:8671. doi: 10.1038/s41598-019-45191-2 (PMC6572822; doi:10.1038/s41598-019-45191-2)
Supplement: Supplementary file 1 — Supplemenrary information [file 41598_2019_45191_MOESM1_ESM.pdf]

**Short exposure to cold atmospheric plasma induces senescence in human skin fibroblasts and adipose mesenchymal stromal cells**

Marion Bourdens, Yannick Jeanson, Marion Taurand, Noémie Juin, Audrey Carrière, Franck Clément, Louis Casteilla, Anne-Laure Bulteau and Valérie Planat-Bénard

*Supplementary information*

**A.**

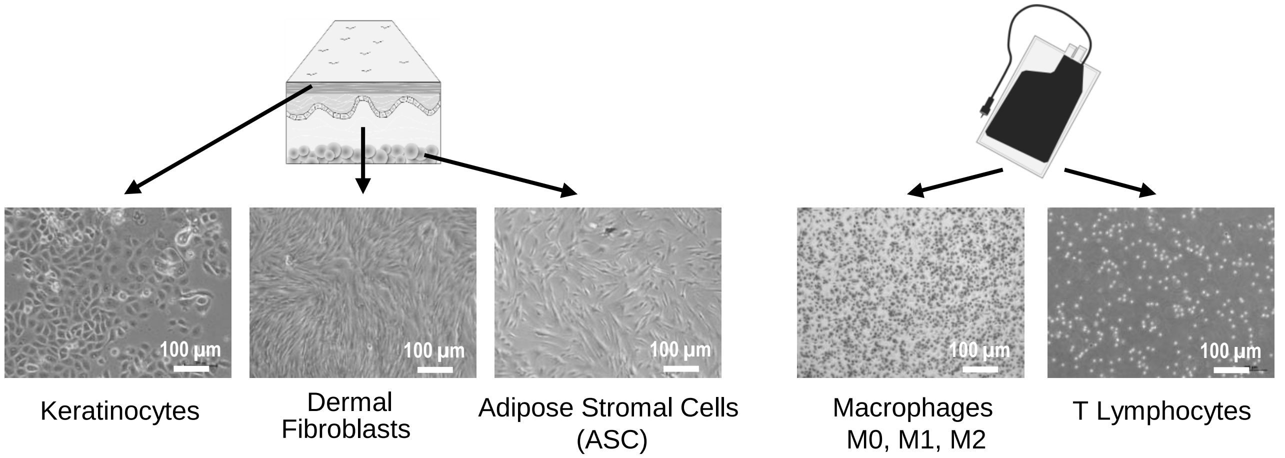


**B.**

| | Keratinocytes | | Dermal fibroblasts | | ASC | | Macrophages | | | | | | T Lymphocytes | |
|---|---|---|---|---|---|---|---|---|---|---|---|---|---|---|
| | | | | | | | M0 | | M1 | | M2 | | | |
| | NT | CAP | NT | CAP | NT | CAP | NT | CAP | NT | CAP | NT | CAP | NT | CAP |
| **Alive** | 91.3 ± 1.5 | 90 ± 1.3 | 95.5 ± 0.7 | 88.8 ± 0.8*** | 93.7 ± 1.1 | 85.6 ± 1.4** | 81.7 ± 2.2 | 82.5 ± 3.3 | 85.5 ± 0.8 | 68.6 ± 9.5 | 78 ± 2.5 | 86.5 ± 3.7 | 86.2 ± 6.8 | 6.8 ± 3.8*** |
| **Apoptotic** | 1.0 ± 0.2 | 0.9 ± 0.1 | 2.2 ± 0.4 | 5.1 ± 0.4* | 2.1 ± 0.8 | 5.1 ± 1.3 | 3.6 ± 1.0 | 3.7 ± 1.5 | 1.2 ± 0.4 | 18.7 ± 7.3 | 3.0 ± 0.5 | 3.8 ± 1.3 | 3.2 ± 3.2 | 2.0 ± 1.9 |
| **Dead** | 7.7 ± 1.4 | 9.1 ± 1.2 | 2.3 ± 0.3 | 6.1 ± 0.6** | 4.2 ± 0.2 | 9.4 ± 2.1* | 14.7 ± 1.5 | 13.9 ± 1.8 | 13.2 ± 0.7 | 12.7 ± 2.9 | 19 ± 2.0 | 9.7 ± 2.4* | 10.5 ± 4.1 | 91.3 ± 2.5*** |

** p<0.05 **p<0.01 *** p<0.001 NT vs. CAP*

**Supplementary Figure 1. Selective sensitivity of various human cells 24 hours after CAP treatment.** (**A**) Freshly prepared human cells from skin sample and blood were treated by CAP He for 3 min in PBS, immediately incubated 1 hour at 37°C before PBS was replaced by fresh culture medium. (**B**) Percentage at 24 hours of alive, apoptotic and dead human cells was obtained as described in Figure 1A for naïve M0, inflammatory M1 and reparative M2 macrophages, T lymphocytes, keratinocytes in addition to dermal fibroblasts and ASC.

| Gene | Forward primer sequence | Reverse primer sequence |
|---|---|---|
| **p21** | GACCTGTCACTGTCTTGTA | CCTCTTGGAGAAGATCAGCCG |
| **p53** | GTGAGCGCTTCGAGATGTTC | ATGGCGGGAGGTAGACTGAC |
| **PPARγ2** | GATACACTGTCTGCAAACATATCC | CCACGGAGCTGATCCAA |
| **LPL** | GGTCGAAGCATTGGAATCCAG | TAGGGCATCTGAGAACGAGTC |
| **Adiponectin** | TGCCCCAGCAAGTGTAACC | TCAGAAACAGGCACACAACTCA |
| **C/EBPα** | GCCGGGAGAACTCTAACTCC | CTGCAGGTGGCTGCTCAT |
| **αSMA** | GAAGAAGAGGACAGCACT | TCCCATTCCCACCATCAC |
| **CTGF** | TGCCCGGGAAATGCTGCGAG | CAGTCGGTAAGCCGCGAGGG |
| **COL1A** | GCTAACCCCCTCCCCAGCCA | GAGCAGGAGCCGGAGGTCCA |
| **Vimentine** | CCTTGAACGCAAAGTGGAATC | GACATGCTGTTCCTGAATCTGAG |
| **MMP1** | ACAGCTTCCCAGCGACTCTA | CAGGGTTTCAGCATCTGGTT |
| **Fibronectin** | CAGTCCCCGGTGGCTGTCAGT | CAGCCCCAACTTTGGTCGGCT |
| **COL3A1** | CCAGGAGCTAACGGTCTCAG | CAGGGTTTCCATCTCTTCCA |
| **Caldesmon** | AGATTGAAAGGCGAAGAGCA | TTCAAGCCAGCAGTTTCCTT |
| **GLUT1** | ATACTCATGACCATCGCGCTAG | AAAGAAGGCCACAAAGCCAAAG |
| **HK2** | GCGGCTCAAGACAAGGGGCA | GGCCACCACAGTGCACACCT |
| **LDHA** | TGGCAGATGAACTTGCTCTTGT | CTTTCTCCCTCTTGCTGACG |
| **MCT4** | ACCCACAAGTTCTCCAGTGC | ATGTAGACGTGGGTCGCATC |
| **MT-ND1** | CCCTAAAACCCGCCACATCT | GAGCGATGGTGAGAGCTAAGGT |
| **β-actine** | AGAGCTACGAGCTGCCTGAC | AGAGCTACGAGCTGCCTGAC |
| **PUM** | AGTGGGGGACTAGGCGTTAG | GTTTTCATCACTGTCTGCATCC |

**Supplementary Table. List of primer sequences**
